# What Is the Essential Basis of Professional Ethics, and the Proper Relation of Trade?

**Published:** 1890-11

**Authors:** J. Morgan Howe


### WHAT IS THE ESSENTIAL BASIS OF PROFESSIONAL ETHICS, AND THE PROPER RELATION OF TRADE?[1]

BY J. MORGAN HOWE, M.D., M.D.S.

"If we define altruism as being all action, which in the normal course of things benefits others instead of benefiting self, then from the dawn of life altruism has been no less essential than egoism."

In the agitation of moral questions, which has progressed for some time, the arguments of those who would raise the standard of ethical requirements the highest are opposed by what their authors probably regard as practical considerations.

The urgency and justice of mutual professional obligations has been offset by the demands of egoism, which in the struggle for existence and preferment cannot be ignored.

It cannot be denied that a desire for adequate remuneration is as fit to be entertained by a professional man as by a man of business, nor, on the other hand, can the proposition be successfully

---

[1] Read before the New York Odontological Society, June 17, 1890.

refuted that many of the devices for gain, which trade employs without reproach, are unbecoming and intolerable when resorted to by members of a profession.

What, then, is the ethical basis on which the details of professional conduct may rest? Men are discussing questions of professional conduct, one side demanding altruism, and the other replying as if there was no reasonableness in the demand.

To raise a standard of professional morals, which requires the highest nobility and unselfishness, does seem inconsistent with the facts presented, that professional men are not necessarily devoid of selfishness, must have the means to live, and not unfrequently desire and obtain the luxuries of life. What, then, is it to be professional, and what are the limitations of the demands of professional ethics?

Whether dentistry is regarded by all who practise it as a specialty of medicine or not, its professional status can only be maintained by such conduct as that or other professions would approve. While dentistry has been making such rapid progress in certain ways, its material prosperity seems to be a hinderance to its moral development, and the battles of thirty years ago are renewed. At that time some of the most advanced thinkers were just beginning to express congratulations on the progress of professional liberality, as shown in the freedom with which information was imparted to professional associates, and congratulations are still often heard at public meetings and clinics that we have still further advanced in making knowledge free.

It seems to be recognized that it was the business or trade spirit which formerly dominated, when dentists would not reveal their methods of practice, nor admit their associates to their offices or laboratories, for fear they would copy their instruments or methods; and that the change that has been wrought has been through the growth of a professional spirit among dentists.

This has stimulated interest in the progressive development of dentistry as a scientific and useful department of the healing art.

It has caused men to make known to their *confrères* what they have found to be an advance on previous methods of practice. Through the influence of professional spirit men have freely given to their associates what they might have withheld for their own exclusive benefit.

Some recipients of information thus given may be in the same town, and competing for the same patronage, as he who makes the discovery or improves on the old method; but altruism prevails over selfishness, so that ideas, methods, and contrivances are made public for the benefit of the brother worker, that he may do better for his patient and for himself. Such actions are clearly opposed to all our ideas of *business*, for if the latter prevailed, such proceedings would not be entertained.

It is the business idea that prompts when one has discovered some specially valuable idea, or devised some contrivance that he regards as valuable, and proceeds to *protect his rights* in his method or invention; he is governed by business instincts in reserving it for his own benefit by a patent, instead of by the method of secrecy, and it is manifest that the trade spirit has again triumphed over interest in the general welfare.

The measure of business influence over individuals varies, and we see some claiming their rights in a sand-paper disk or a matrix, while others would have restrained their trade instincts perhaps till claims of greater magnitude presented. But whatever the point at which it is decided that an idea or device is worth too much to bestow freely to the common fund for the common good, business triumphs then and there over the interest all professional men should take in helping their *confrères.*

Without in the least disparaging business principles or the devices of trade, by which men seek advantage over business competitors, general admiration of the fraternal spirit manifested in professional actions is evident. This is clearly shown in the contentions of those patentees who are loath to have any of their actions regarded as unprofessional. Although they have a perfect right to what is their own, to give, to withhold, or to sell for lucre, if they choose either of the latter, they surely have no cause of complaint if their associates fail to concede to them the honor of being controlled by a professional spirit in those things, the monopoly of which they choose to retain for their own advantage, or sell to manufacturers who do the same; whether these mercenary advantages are sought to be maintained by means of secrecy or by the aid of patent laws.

The better element in all so fully recognizes the justice and nobility of professional sentiment, on the part of a body whose every member is debtor to the accumulated industry, talent, and experience of the past, that men whose pockets jingle with the coin of trade, in articles or ideas of supposed value to their associates, have their vision obscured in the conflict between their nobler and more sordid tendencies.

They argue and contend and apparently befog the question of their right, both to the aforesaid *pelf* and to the *honor* of denying themselves the advantage of its possession.

Neither true professional feeling nor ethics requires professional men to abrogate their desire for full remuneration for faithful services to their patrons. And those are mistaken interpretations which so construe what has been written by those who have advocated high professional standards.

The highest qualifications in any vocation are worthy of adequate remuneration, and he serves his profession as well as benefits himself who, by the application of the highest skill and integrity, gets such appreciation of his abilities as enables him to demand large fees; for he thereby enables his *confrères* also to obtain better remuneration, and leaves more for those to do who are willing to accept less.

Repression of charlatanry and fraud practised on the public has always been aimed at by professional men, but the essence of professional spirit is to be found in fraternal relations established and maintained among members of the same calling. This implies a minimizing of business instincts and methods in all the mutual relations of professional intercourse. This seems to have been recognized in all codes of ethics.

The interdiction of public advertising—which is so compatible with trade methods—and provisions against one member of a profession in his intercourse with the patient of another, making suggestions calculated to weaken confidence in his associate, have evidently been adopted, because it was recognized by the framers of such codes that unless solidarity was cultivated among members of a profession, a professional status could not be maintained.

It is for the maintenance and extension of this idea of a community of interest in the knowledge and experience of the whole body that colleges, societies, and professional literature must exist, or they have no good reason for being; and the general progress of any profession, as such, will necessarily be measured by the freedom with which all the knowledge and means to improved practice can be acquired by those members having the least resources; just as communities and nations are elevated in the scale of civilization in proportion as the least educated and poorest of the people, are lifted up morally and materially.

The progress of a profession, as well as a nation, is to be measured not by the condition of the highest but of the lowest classes.

If this be not true, why should we not go back to the practices of the days of professional infancy, when those most competent in any particular kept their secrets or sold them for money?

Why should societies, such as this, hold meetings where incidents, appliances, methods, and principles are presented and discussed for the common good of those present, and afterwards are published for a larger audience to read and appropriate, unless dentistry is to progress thereby, not alone in its front ranks, but also among its least advantaged practitioners? What reason is there for the free dissemination of such knowledge that does not apply to every proprietary secret or patented contrivance?

Those who freely contribute—without thought of return—according to their ability for the general good are nevertheless not without their reward,—the stimulation and development of their own powers, the consciousness of doing their part, and the appreciation and esteem of their associates. In other professions, men who become recognized as having special ability are called in counsel and obtain special fees; in dentistry this is less common, mainly, it would seem, because there is less general assurance of mutual professional feeling and more suspicion of trade spirit. Those who contend for their right to money remuneration for their inventions seem to help produce such an impression. It is a business right which has no place in the mutual relation of professional associates. They may obtain the reward they seek, but they have sold their birth-right. These gentlemen have allied themselves with manufacturers and dealers in supplies, and ought not to seek both the profits and emoluments of the latter, as well as the favor of professional recognition.

The business of making and selling goods for the use of professional men is an honorable one, but certain business principles and methods prevail that separate it widely from a profession, and make its interests dissimilar, and in some respects antagonistic to the profession to which it caters.

Dental manufacturers and dealers and those dentists who sell them their patents, or secret formulæ, have used the argument that the prospect of money recompense is the only incentive that can be relied on to insure progress. Their interests in their own benefits, arising from the cultivation of this sentiment, make them overlook the fact that the best and most valuble work in the world has always been done without regard to recompense; although the result of such work has often been appropriated by others, or turned to their practical use. Dwinelle devised and suggested our use of the screw, and Jack gave us the matrix, but others for slight modifications of these ideas sought pecuniary reward by patents.

Dr. Stevens, an eminent oculist, has, within a short time, given his professional associates the phorometer, an instrument which represents more originality than any patented thing now used by dentists; and his failure to patent illustrates the professional idea as opposed to trade principles. Possibly some optician might be glad to have the monopoly of its manufacture, and pay well for the privilege; but hardly an oculist could be found to admit that such an arrangement would tend to professional progress. On the contrary, progress in a professional sense is checked by patents to the extent that the ideas or inventions are covered by the law; improvements are either prevented as infringements, or else are smothered by those interested in the original patent.

It is against the interests of manufacturers to let improvements get on the market. The refusal or failure of the Bell Telephone Company to improve the instruments used by the public has recently been discussed in the public press, and the New York *World* comments thus: "The question naturally is, Why is not the public given the benefit of these improvements? The answer is perfectly plain, Because it would cost the monopoly something to supply new instruments. It is cheaper for the original patentees to buy up and pigeon-hole all outside improvements, or to refuse a license for their use, than to accommodate the public;" and adds that it is not easy to suggest a practical remedy for this state of affairs.

Professional wants in the line of improvement are antagonistic to the same commercial factors which elect that the public shall have just as much—and no more—as manufacturers and patent owners can make the most out of, and instances are not wanting in which dental manufacturers have bought patents for appliances calculated to supersede those made by them, for the purpose of preventing their appearance in the market.

Such like business expedients, although quite compatible with ordinary trade methods, are entirely at variance with the ethics of a profession, and clearly illustrate a distinct and radical difference between business and professional ethics.

Professional men recognize privileges and duties in their relations to their *confrères* which are not in the line of business, but are distinctly altruistic, and the history of dentistry, as a profession, shows that the beginning of such recognition of the mutual relation of its practitioners was the beginning of professional development.

The dentist of the olden time, who cherished his secret, had the same reasons for doing so that the patentee to day has for asserting his rights in his invention or method, and both exert inhibitory influence on professional growth.

These considerations suggest the question, whether abnegation of self interest, in response to the sentiment of solidarity, is not the basis of professional ethics? Without such feeling a body of men would not be professional, no matter what the sphere of their work.

It seems to be entirely due to such sentiment that men begin to take interest in raising educational standards, disseminating knowledge, and generally interesting themselves in helping their *confrères* to do more and better work. Without such feeling we return to secrecy and isolation.

If these are fair and true statements, is there a point at which professional men can consistently withhold what they have acquired from their brother? No, there is no such point.

Complete solidarity in matters of common interest is the essential basis of professional ethics, and with this generally admitted and practised, all other ethical questions would be subsidiary. This draws a distinct line between the professional status and trade. Manufacturers and business-men keep their factory- and office-doors closed against their competitors, instead of seeking to benefit and enlighten those in the same business. When they combine for mutual advantage, it is not to help the weak, nor to disseminate information that might help others to do better, but to exclude from the advantage of combination those who are powerless to compel admission, or are unwilling to submit to business dictation; to accomplish by uniting what they cannot do separately, in the direction of monopoly.

Many dental manufacturers claim a certain sort of professional standing because they have been dental practitioners, or hold diplomas. They attend society meetings and clinics to show their wares in a quasi-professional way, and not infrequently read papers and perform clinics with more or less business suggestions. These conditions have met with but little open opposition, perhaps because to some the trade atmosphere thus introduced was not unpleasant, and partly through unwillingness to incur the disfavor of the business interests represented, the latter being jealous of their opportunities and aggressive in maintaining their influence.

It is an open secret that some prominent men have been the recipients of benefits—more or less direct and material—from dealers and manufacturers, and that the sentiment of professional circles in which these men move seem to be affected concurrently. A large number of the manufacturers own and publish journals which contain the writings of dentists and current society reports, and are the special medium of advertising of the house owning them. A periodical literature of dentistry has in this way been largely monopolized by the dental trade, dentists themselves furnishing the means. The most obvious expression of dissatisfaction with existing conditions has been made, in various efforts, during the past ten years, to establish journals of professional literature that should not be under the control of manufacturing firms; and more recently in individual utterances of disapproval. As a result of the latter, the men who have been sufficiently interested in the professional status of dentistry to call attention to the insidious influence and control which is exerted by manufacturers and dealers have their motives impugned and themselves maligned by those who think their material interest jeopardized; but tradesmen, as well as professional associates, must recognize the fact that self-interest would not prompt men to write against these abuses, as Drs. Meriam, Jack, and Smith have done. Nothing but a sense of need, and willingness to perform a duty, could have induced their action. The thanks of their associates have been meagrely expressed, while some have not been reticent in pointing out what they considered flaws in the indictment. But appreciation has not been absent, though unexpressed, and the attitude of the journals owned by business-houses has demonstrated the need of independence in journalism, as well as in other relations.

Those are erroneous ideas of professional progress that favor a commercial atmosphere, or that would obtain an advantage at the price of placing professional men or organizations under obligations to those seeking patronage. It is, for instance, commonly reported that some dental colleges are the regular recipients of gratuities of all the artificial teeth they use in their infirmaries, and that the students are recommended to make their purchases of the firm making such gifts.

The relations of societies to manufacturing concerns are also often of a nature to compromise professional dignity. In New York, one of the societies accepted for years the free use of a dental depot for its regular meetings, and also for its monthly clinics. This may seem to have a justification in the saving of expense to members, but the anomalous nature of such arrangements would appear to have been recognized when, some time ago, after some quiet agitation, the society decided to change its place of meeting, relinquishing this much of the bounty; but the free use of the salesroom has been retained till the present time for the regular clinics. These clinics have undoubtedly been productive of much benefit. Their interesting character and efficiency was for years largely due to the untiring labors of Dr. C. F. W. Bödecker, chairman of the Clinic Committee. Some three years ago, on his withdrawing from active interest in the society, the writer asked him the reason for such action, and was informed that one of the reasons was that his course in sometimes introducing at the clinics appliances and devices other than those made by their host, which he considered of interest, met with objection and remonstrance from those in charge of the depot. The statement is made that the invitations and programmes are printed gratis by the business-house, and material for the operators furnished without cost. To these it may be added that the same society has accepted, on several occasions, donations of considerable sums from the same business concern, to help defray the expenses of special large meetings, and on at least one of the special occasions the same business-house was given the sole right to make an exhibit of goods in the place of meeting. One of the Brooklyn societies has also been under some obligations to a dental supply house for use of rooms, I am informed. How many organizations throughout the country may have business arrangements of a like character for professional advancement is not at present known? These are cited merely as illustrations, and if there is anything stated amiss the writer will be glad to be corrected. But the question is raised whether these things tend to professional growth, or whether all that may be gained is not more than counterbalanced by loss in other ways? When the State of Louisiana would have been benefited by larger supplies of money to defray the expense of repairing the levees against the rising floods, Governor Nichols thought the advantage would be gained at too great a cost if the people of the State were placed under obligations to the Louisiana Lottery Company, and therefore returned their check for one hundred thousand dollars, which was tendered as a gift.

The business interest in obstructing improvements under certain conditions, and restricting freedom of information, is shown also in the present condition of manufacture and sale of proprietary and secret compounds. The progress of dentistry demands that its practitioners should know exactly what they are using, whereas the extensive advertisement and sale of proprietary medicaments, cements, and alloys shows that the majority must have only a very vague idea of the formulæ of such compounds. It is for the apparent interest of the trade that these things should be so, and dentistry has developed so rapidly, and is yet so young, that it could not be expected that abuses would not also grow up.

But it is quite evident that the time has arrived when such conditions are recognized as opposed to scientific progress, and, being seen, should be corrected by the determination of individuals to use only those compounds whose formulæ are made public.

Sir Joseph Lister, in a recent address before the Medical Society of London on "A New Antiseptic Dressing," took occasion to compliment and thank those firms of manufacturing chemists and pharmacists who aided him in his chemical researches, and assisted him by supplying the needed material. It is to be observed, in the report of his address, that the chemists freely informed him of their methods and the result of their experiments, without reserve or secrecy. It may well be doubted whether this eminent surgeon or any other self-respecting physician would have felt called upon to thank manufacturers or commend their products if the formula of the dressing had been a secret. It is safe to say that a secret preparation would not have been used, much less made the topic of an address. Pharmacists are publishing the formulæ of their preparations as an inducement to physicians to favor their use, and there are similar reasons why dentists should require the same inducements. Dental pharmacy ought to be a subject of scientific study and practical experiment and comparison, instead of being left in hands as secret and mysterious as the alchemists of old.

Manufacturers have all these years been trading on the assumed ignorance or indifference of dentists, as is shown in the method of advertising their compounds under proprietary names, with more or less impossible virtues. Alloys are presented as "rich in precious metals," or as containing gold and platinum, in deference to supposed benefits, or as an excuse for high prices, but other elements in their proportions kept secret, just as quack nostrums are advertised to the public.

Considerable attention has been given to the question of the relative value of ingredients of cements and alloys by individual dentists, but there is every reason for believing much progress may yet be made, and nothing would tend more to improvement than a general and familiar knowledge of just what is used, and comparison of results. Such topics are as legitimate and necessary subjects for society discussions as any others.

Manufacturers are not necessarily to be condemned for pursuing a business policy in regard to matters left in their hands by our own indifference and preoccupation. We need only to recognize what is needed for the advance of scientific and professional interest, and be governed thereby. But we are confronted by the fact that dental purveyors claim a professional standing as dentists, own journals, and resent restriction of their claims, and even the discussion of these subjects.

If the conclusion that community of interest in matters of common professional concern is the essential basis of professional ethics, manufacturers and dealers in dental goods surely have no claim to such recognition. But it is unnecessary that our interpretation of ethics should furnish the ground for denial of such a claim, the general attitude of the majority of manufacturers, shows plainly a very different sort of interest in dentistry from that of its practitioners, as does also the combination they have formed—for their own interest, and we claim against ours—to limit competition and exclude from the facilities of business relations those who are for any reason not of their coterie.

Further justification for refusing to accord professional standing to these so-called "professional business-men" seems unnecessary, but the editor of their leading journal has furnished it, in discussing these very questions. In the same article in which statement is made of the circumstances and honors attending his obtaining the M.D. and D.D.S. degrees, it is also stated that an allegation made in Dr. Jack's "Saratoga paper," "is calculated, if not intended, to damage the business of the company publisher of the *Dental Cosmos*," and he is accordingly *dared* to make his allegations more specific, and in that case, the editor says, he "will give him [Dr. Jack] the opportunity to justify himself . . . in a defence in open court." Nothing, perhaps, could more clearly show the difference that must necessarily exist between the professional and the business instinct, and the incongruity of the claim to professional standing of those engaged in the business of dental supplies, than this threat of a lawsuit against Dr. Jack.

Our relations with the firms and individuals who supply our instruments and materials ought to be entirely amicable, but we cannot be under obligations to them without recognizing a claim—either expressed or implied—to some return. The dental trade should occupy the same relation to dentistry as do the purveyors of supplies to other professions,—namely, caterers to their needs.

If dental manufacturers should progress to professional relations among themselves, it seems quite possible that they would in some ways be benefited. At present their relations both to each other and to their patrons seem to be of a purely business character. With this relation we could find no fault, provided its business character was fully recognized by both sides. Business relations may be none the less friendly because so recognized, and professional relations are not necessarily more friendly, but are essentially different. Progress seems in no small degree to depend on a fuller recognition of what these differences are, and with a desire to stimulate thought and discussion this paper is submitted for your criticism.

